# The Brief Case: *Cystoisospora belli* in the duodenal aspirate of an HIV-infected child

**DOI:** 10.1128/jcm.01765-25

**Published:** 2026-06-10

**Authors:** Nidhi Tejan, Nidhi Bhatnagar, Anju Dinkar, Shikha Singh

**Affiliations:** 1 Department of Microbiology, Sanjay Gandhi Post Graduate Institute of Medical Sciences 30093, Lucknow, Uttar Pradesh, India; 2 Department of Microbiology, Hind Institute of Medical Sciences, Barabanki, Uttar Pradesh, India; 3 Department of Pediatrics, Government Medical College, Haridwar, Uttarakhand, India; Mayo Clinic Minnesota, Rochester, Minnesota, USA

**Keywords:** chronic diarrhea, HIV infected, immunocompromised, *Cystoisospora belli*

## CASE

A 10-year-old boy presented with a history of recurrent, large-volume, watery loose stools occurring multiple times a day, accompanied by progressive abdominal distension, intermittent low-grade fever, and significant unintentional weight loss for the past 2 years. The diarrheal episodes were non-bloody, and there was no history of passage of mucus, suggesting a non-inflammatory etiology. His appetite had gradually decreased, and his caregivers reported increasing fatigue and reduced activity levels.

On physical examination, the child appeared pale and malnourished. Notably, oral candidiasis was present, along with generalized lymphadenopathy involving cervical and axillary nodes. Systemic examination did not reveal organomegaly, but signs of dehydration and pallor were evident. Laboratory investigations showed hypochromic microcytic anemia, with a hemoglobin level of 8.0 g/dL (reference range for age is 13.5 ± 2 g/dL), consistent with chronic illness and nutritional compromise. Five serial stool specimens collected on separate days were examined using direct wet mount, formalin–ether concentration, and modified acid–fast staining on both direct and concentrated specimens; all were unremarkable, with no parasitic forms detected.

Given the chronic diarrhea and clinical suspicion of malabsorption, an upper gastrointestinal endoscopy with duodenal biopsy and aspirate examination was performed. Histopathological evaluation of the duodenal mucosa showed largely preserved villous architecture, with occasional mild villous flattening and non-specific inflammatory changes. No parasitic stages were identified. No special stains were performed on tissue sections, as routine histological examination did not reveal suspicious structures.

**Fig 1:**
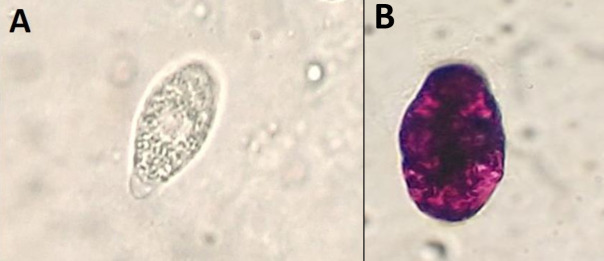
(**A**) *Cystoisospora belli* oocyst in unstained preparation of duodenal aspirate at 400× magnification. (**B**) Modified Kinyoun staining of duodenal aspirate showing acid–fast elliptical oocyst under 1,000× magnification.

Microscopic examination of the unstained duodenal aspirate revealed numerous elliptical oocysts morphologically consistent with *Cystoisospora belli* (Fig. 1A). Modified Kinyoun acid–fast staining was subsequently performed as a confirmatory test, demonstrating acid–fast elliptical oocysts characteristic of *Cystoisospora* species (Fig. 1B). Modified acid–fast staining is not routinely applied to duodenal aspirates unless parasitic structures are suspected on direct microscopy.

Further evaluation for underlying immunosuppression revealed esophageal candidiasis on endoscopy, and human immunodeficiency virus (HIV) testing was positive by three ELISA kits, confirming underlying HIV infection contributing to his chronic presentation. Oral and esophageal candidiasis were considered supportive indicators of immunosuppression. His CD4 count was 150 cells/mm^3^.

Based on these findings, a diagnosis of cystoisosporiasis in an HIV patient was established. He was started on antiretroviral therapy. The child was treated with 250 mg trimethoprim–sulfamethoxazole administered in two divided doses for 10 days. He showed notable symptomatic improvement, with reduction in stool frequency and gradual weight gain.

## DISCUSSION

*Cystoisospora belli* is an obligate intracellular coccidian protozoan that infects the epithelial cells of the small intestine. It is one of the few coccidian parasites known to cause human disease, along with *Cyclospora* species (formerly grouped as *Cyclospora cayetanensis*). The organism is found worldwide, particularly in tropical and subtropical regions, where it is associated with sporadic and opportunistic infections. In immunocompetent hosts, infection may cause self-limited diarrhea, but in immunocompromised individuals—especially those with advanced HIV infection—it can lead to chronic, relapsing, and sometimes life-threatening diarrhea (1).

Infection is acquired through ingestion of mature oocysts containing two sporocysts, each with four sporozoites, from contaminated food or water. Once inside the small intestine, sporozoites excyst and invade the epithelial cells. They undergo both asexual (schizogony) and sexual (gametogony) replication cycles, producing oocysts that are shed in feces. These oocysts require 24–48 hours in the environment to sporulate and become infective (2).

The parasite causes damage to the intestinal mucosa by invading epithelial cells, leading to villous atrophy, epithelial desquamation, and mild inflammation of the lamina propria. The resulting malabsorption contributes to chronic diarrhea, abdominal pain, anorexia, and weight loss. In HIV-infected individuals, the severity of infection correlates with immune suppression, particularly when CD4^+^ T-cell counts fall below 200/µL (3).

Symptoms typically include prolonged watery diarrhea, abdominal cramps, anorexia, malaise, and significant weight loss. Fever may be present but is usually low grade. Dehydration and malnutrition can occur in chronic cases. Extraintestinal manifestations are rare but may include infection of the biliary tract and mesenteric lymph nodes (1).

In immunocompetent individuals, infection may resolve spontaneously within 2–3 weeks, whereas in HIV-infected patients, it can persist or relapse if not adequately treated or if immune reconstitution does not occur with antiretroviral therapy (3).

## DIAGNOSIS

Diagnosis of *C. belli* infection is primarily made by microscopic detection of oocysts in stool specimens using wet mounts or special stains. However, oocyst shedding may be intermittent and low in number, leading to false-negative results. Concentration techniques, such as formalin–ethyl acetate sedimentation or Sheather’s sugar flotation, improve detection but do not fully overcome these limitations.

Oocysts are large (25–30 μm × 12–19 μm), ellipsoidal, and transparent, with a smooth double-layered wall. Immature oocysts contain one sporoblast, while mature ones contain two sporocysts. Modified Ziehl–Neelsen or Kinyoun acid–fast staining renders the oocysts pink to red against a blue background. Autofluorescence under ultraviolet light microscopy can also aid identification (2).

In this case, repeated stool examinations were negative, emphasizing that *C. belli* may be missed if only fecal samples are examined. Duodenal biopsy and duodenal aspirate examination are both recognized diagnostic options for *Cystoisospora belli* infection, and either specimen may demonstrate parasitic stages in situations where the other is non-diagnostic, as illustrated in the present case and reported in the literature. Histopathological examination of duodenal biopsy specimens may reveal intracellular parasitic stages within epithelial cells; however, detection can be limited by sampling variability. In contrast, duodenal aspirate examination allows direct visualization of oocysts within the intestinal lumen. Detection of oocysts in duodenal aspirates represents active intestinal infection and is of particular clinical significance in immunocompromised pediatric patients, in whom delayed diagnosis may contribute to prolonged diarrhea, malnutrition, and growth failure (4).

## DIFFERENTIAL DIAGNOSIS

The main differentials include other coccidian parasites causing chronic diarrhea in immunocompromised hosts:

- *Cryptosporidium* species: 4–6 μm diameter, spherical oocysts, acid–fast.
- *Cyclospora* species: 8–10 μm diameter, spherical, variably acid–fast, autofluorescent.
- *Sarcocystis* species: oocysts/sporocysts may be detected in stool, less commonly implicated but should be considered.
- Microsporidia: spores (1–2 μm), demonstrated with modified trichrome stain.

Accurate differentiation is crucial because treatment regimens differ (2).

## TREATMENT

The drug of choice for *C. belli* infection is trimethoprim–sulfamethoxazole (co-trimoxazole). Standard therapy consists of 160 mg/800 mg (trimethoprim–sulfamethoxazole) twice daily for 7–10 days in adults, with weight-adjusted dosing in children. In this case, the patient received an age-adjusted standard therapy with a good response.

For patients intolerant to sulfonamide drugs, pyrimethamine with leucovorin may be used as an alternative. In HIV-infected individuals, prophylactic cotrimoxazole for *Pneumocystis jiroveci*i pneumonia also protects against *C. belli* infection. Relapse is common if the immune status does not improve; hence, antiretroviral therapy is essential for long-term control (3).

## EPIDEMIOLOGY AND PUBLIC HEALTH SIGNIFICANCE

*C. belli* infection is globally distributed but more prevalent in developing regions where sanitation is poor. Reported prevalence in HIV-infected patients with diarrhea ranges from 2% to 10%. Transmission occurs through the fecal–oral route, and sporulated oocysts in contaminated food or water serve as the source of infection. Direct human-to-human transmission has not been demonstrated, as oocysts are shed unsporulated and require environmental sporulation to become infective, making person-to-person spread unlikely, compared with *Cryptosporidium* (3).

The detection of oocysts in duodenal aspirate adds to existing literature by demonstrating that aspirate examination can substitute for biopsy in resource-limited settings. The finding underscores the importance of simple wet mounts in diagnostic parasitology.

## CONCLUSION

*Cystoisospora belli* infection should be considered in HIV-infected patients presenting with chronic diarrhea, especially when stool and biopsy results are inconclusive. Duodenal aspirate microscopy is a simple, effective diagnostic tool that can reveal parasitic stages missed by other methods. Prompt treatment with cotrimoxazole leads to rapid clinical improvement, and awareness of this diagnostic approach can significantly aid patient management in resource-constrained settings.

## SELF-ASSESSMENT QUESTIONS

1. Which of the following specimens cannot be used for the diagnosis of cystoisosporiasis?
  - Stool specimen.
  - Duodenal biopsy.
  - Duodenal aspirate.
  - Blood specimen.
2. Which staining method is most appropriate for demonstrating *Cystoisospora belli* oocysts?
  - Giemsa stain.
  - Modified Kinyoun acid–fast stain.
  - Silver methenamine stain.
  - Trichrome stain.
3. Which of the following statements about *Cystoisospora belli* infection is TRUE?
  - It causes self-limited diarrhea in most HIV-infected patients.
  - Direct person-to-person transmission is common.
  - Cotrimoxazole is the treatment of choice.
  - The oocysts are spherical and measure 4–6 μm.

## ANSWERS TO SELF-ASSESSMENT QUESTIONS

1. Which of the following specimens cannot be used for the diagnosis of cystoisosporiasis?
  - Stool specimen.
  - Duodenal biopsy.
  - Duodenal aspirate.
  - Blood specimen.

Answer: d. *Cystoisospora belli* primarily infects the intestinal epithelium, and diagnosis is based on detection of oocysts in stool or occasionally in duodenal aspirates or biopsy specimens when stool examinations are negative. Blood specimens are not useful, as the parasite does not circulate in the bloodstream.

1. Which staining method is most appropriate for demonstrating *Cystoisospora belli* oocysts?
  - Giemsa stain.
  - Modified Kinyoun acid–fast stain.
  - Silver methenamine stain.
  - Trichrome stain.

Answer: b. *C. belli* oocysts are acid–fast and appear pink to red on modified Kinyoun staining, which is the diagnostic stain of choice.

1. Which of the following statements about *Cystoisospora belli* infection is TRUE?
  - It causes self-limited diarrhea in most HIV-infected patients.
  - Direct person-to-person transmission is common.
  - Trimethoprim–sulfamethoxazole is the treatment of choice.
  - The oocysts are spherical and measure 4–6 μm.

Answer: c. Trimethoprim–sulfamethoxazole effectively treats *C. belli* infection and prevents relapse. Oocysts are large, ellipsoidal (25–30 μm), and transmitted via contaminated food or water.

**TAKE HOME POINTS**

1. Negative stool microscopy does not rule out *Cystoisospora* infection; oocyst excretion can be intermittent
2. Although duodenal aspirate and biopsy may yield important diagnostic findings, these invasive procedures should be considered only in carefully selected patients with persistent clinical suspicion following multiple negative stool examinations.
3. Simple staining methods (Kinyoun or Ziehl–Neelsen) can reliably identify the organism.
4. Trimethoprim–sulfamethoxazole remains highly effective, but relapse may occur if immune reconstitution is poor.

## References

[1] Kurator K, Mason L, Bruckner D, Tan H, Mathisen G. 2025. Diagnosis of cystoisosporiasis in a patient with HIV and modestly decreased CD4 counts. IDCases 40:e02259. doi:10.1016/j.idcr.2025.e02259 40511254 PMC12159879

[2] Centers for Disease Control and Prevention (CDC). 2024. Cystoisosporiasis: parasite biology and laboratory diagnosis. *In* DPDx – laboratory identification of parasites of public health concern. CDC, Atlanta (GA). https://www.cdc.gov/dpdx/cystoisosporiasis/.

[3] Frickmann H, Sarfo FS, Norman BR, Agyei MK, Dompreh A, Asibey SO, Boateng R, Kuffour EO, Blohm M, Di Cristanziano V, et al.. 2025. Epidemiological, clinical, and immunological features of Ghanaian people-living-with-HIV (human immunodeficiency virus) and molecular proof of Cystoisospora belli in their stool samples. Pathogens 14:212. doi:10.3390/pathogens14030212 40137697 PMC11944657

[4] Murphy SC, Hoogestraat DR, Sengupta DJ, Prentice J, Chakrapani A, Cookson BT. 2011. Molecular diagnosis of cystoisosporiasis using extended-range PCR screening. J Mol Diagn 13:359–362. doi:10.1016/j.jmoldx.2011.01.007 21458380 PMC3077734

